# Socioeconomic patterns of underweight and its association with self-rated health, cognition and quality of life among older adults in India

**DOI:** 10.1371/journal.pone.0193979

**Published:** 2018-03-07

**Authors:** Y. Selvamani, Pushpendra Singh

**Affiliations:** 1 Department of Development Studies, International Institute for Population Sciences (IIPS), Mumbai, Maharashtra, India; 2 Department of Humanities & Social Sciences, Indian Institute of Technology Roorkee, Roorkee, Uttarakhand, India; Medical University of Vienna, AUSTRIA

## Abstract

**Background:**

Underweight defined as body mass index (BMI) < 18.5 is associated with negative health and quality of life outcomes including mortality. Yet, little is known about the socioeconomic differentials in underweight and its association with health and well-being among older adults in India. This study examined the socioeconomic differentials in underweight among respondents aged ≥50 in India. Consequently, three outcomes of the association of underweight were studied. These are poor self-rated health, cognition and quality of life.

**Methods:**

Cross-sectional data on 6,372 older adults derived from the first wave of the WHO’s Study on global AGEing and adult health (SAGE), a nationally representative survey conducted in six states of India during 2007–8, were used. Bivariate and multivariate regression analyses were applied to fulfil the objectives.

**Results:**

The overall prevalence of underweight was 38 percent in the study population. Further, socioeconomic status showed a significant and negative association with underweight. The association of underweight with poor self-rated health (OR = 1.60; *p* < .001), cognition (β = –0.95; *p* < .001) and quality of life (β = –1.90; *p* < .001) were remained statistically significant after adjusting for age, sex, place of residence, marital status, years of schooling, wealth quintile, sleep problems, chronic diseases, low back pain and state/province.

**Conclusion:**

The results indicated significant socioeconomic differentials in underweight and its association with poor self-rated health, cognition and quality of life outcomes. Interventions focussing on underweight older adults are important to enhance the overall wellbeing of the growing older population in India.

## Introduction

Despite rapid economic growth in India in recent decades, the nutritional status of the adult population remains poor [1]. On the one hand India is experiencing a steady rise in the prevalence of overweight and obesity [2], which is globally recognized as a strong predictor of all-cause mortality and chronic diseases such as diabetes, cardiovascular diseases and hypertension. On the other hand, India has the highest number of underweight adults in the share of global adult underweight [2, 3]. The coexistence of undernutrition and overnutrition is referred as the dual burden of malnutrition, which commonly occurs in developing countries and poses a major public health challenge [4, 5, 6, 7, 8, 9].

Both underweight and overweight are associated with increased risks of poor health and mortality. The potential health risks owing to overweight and obesity in western countries are well documented. Further, in recent years, many studies have investigated the health risks of over-nutrition across the globe. Overweight and obesity are strongly associated with all-cause mortality [10, 11, 12], chronic diseases [13, 14], disabilities, poor self-rated health [15, 16, 17, 18, 19, 20], poor health-related quality of life and cognition [21, 22, 23].

Little is known about the nature and patterns of underweight and its subsequent health implications. Globally, undernutrition contributes 16 percent to the disability-adjusted life year (DALY) [24]. Further, the impact of lower body mass index on health is stronger in developing countries. Lower body mass index is associated with increased risk of excess mortality [11, 25, 26], poor cognition [27], poor self-rated health and health-related quality of life [28, 29]. While 35 percent older adults aged 50 and above in India are underweight [30], the association between underweight and subsequent health outcomes is less known in India. A few studies examined the association of low body mass index with mortality [25, 26], poor self-rated health [31] and morbidities [32]. However, these studies used micro-level, hospital-based, region-specific data sets. Also, most of them examined the pattern of undernutrition in India focussing on the younger population (15–49). The extent of undernutrition among the older population has been less documented. Therefore, it is less clear to what extent underweight differs by socioeconomic status among older adults in the country.

Using the SAGE data, the present study examined the socioeconomic pattern of underweight among older adults in India. Further, the association of underweight was examined with three outcomes: self-rated health, cognition and quality of life. Previous literature from India among middle-aged and older populations demonstrates that underweight is concentrated in adults of low socioeconomic status [9, 32, 33, 34, 35, 36, 37] and rural residents [38, 39, 40]. Despite the fact that overweight and obesity are strong predictors of mortality in other parts of the world, in Asian countries such as India and China excess mortality owing to underweight is seen higher [25, 41, 42, 43].

In this context, understanding the association of underweight with self-rated health and other outcomes will provide a better insight into the health implications of underweight and will be useful in policy perspectives. To our knowledge, there is no study which has examined the association of underweight with various health outcomes in India. The outcome variables—poor self-rated health, measures of cognition and quality of life—that were employed in the present study are frequently used in aging and epidemiological surveys. Existing literature strongly suggests that the measure of self-rated health is considered as a global measure of overall health which is strongly associated with other health outcomes such as disability and mortality [28, 44, 45]. Further, measures of cognition and quality of life are important components of physical well-being and major contributors to the overall well-being of the older population [46, 47, 48, 49].

We used nationally representative data on 6,372 Indians aged 50 and above for this analysis which was collected during 2007–8 from six states of India. As the share of the older population in India is growing faster, mainly due to the decline in fertility and mortality, it is necessary to understand the contribution of nutrition-related health implications, which will help policymakers to identify and improve the overall health and well-being of the growing older population in India.

## Materials and methods

### Data

The present study used the first wave data set of the SAGE survey conducted between the years 2007 and 2008. This was the first longitudinal study carried out by the WHO on health and ageing in multiple low- and middle-income countries including India. The target population in the SAGE survey was individuals above 18 years of age. A multistage, stratified clustered sample design was used homogeneously in all countries included in the SAGE. The survey comprised nationally representative samples and yielded results that are comparable to those of similar ageing surveys in high-income countries. This is the latest available data which includes detailed information on health behaviour, use of health services and health outcomes along with a varied set of socioeconomic items from a nationally representative household population aged above 50 in India.

The total sample for the SAGE survey was 12,198. However, we considered the data of 6,372 older adults aged ≥50 in India. The survey encompassed a wide range of geographic and socioeconomic variabilities. Face-to-face interviews were conducted to collect information about the physical characteristics of the dwelling/household as well as to develop a household roster, including sex, age, education, marital status and care needs of each household member. The health status of individuals was also assessed with cognition, quality of life and other tests. A short set of cognition tests measured concentration, attention and memory, which provides an estimate of cognitive ability and its impact on health status (for example, dementia). In biomarker components, various tests were conducted to assess the prevalence of chronic diseases. Further, physical tests and biomarker measures such as walking speed, lung function test and grip strength were conducted. A detailed description about the survey is given in Kowal et al. 2012 [50].

#### Ethics and consent

The SAGE study was approved by the Ethics Review Committee, World Health Organization, Geneva, Switzerland and the Institutional Review Board, International Institute of Population Sciences, Mumbai, India. Informed written consent was obtained from each participant, who were ensured that data would remain confidential and used for research purposes only.

### Methods

#### Body mass index (BMI)

BMI Calculation: BMI was calculated as weight in kilograms divided by height in metres squared. In this study, objectively measured height and weight were used to calculate BMI. Further, it was categorized as < 18.5 kg/m^2^ (underweight), 18.5–24.9 kg/m^2^ (normal weight), 25.0–29.9 kg/m^2^ (overweight), 30.0+ kg/m^2^ (obesity).

#### Measures of socioeconomic status

In this study years of schooling and wealth quintile have been included as the measures of socioeconomic status to examine their association with underweight. Years of schooling was categorized as ‘no schooling’, ‘1–5 years’, ‘6–9 years’ and ‘10 years or above’. A composite wealth index was generated based on household ownership of assets. Principal component analysis was used to create the composite index and categorized as first (lowest), second, third, fourth and fifth (highest) with cut-off points of 20% quintile each [51]. List of variables used to construct wealth quintile is provided in the supplementary file (see S1 File).

#### Self-rated health (SRH)

This study used self-rated health as one of the outcome variables. In SAGE, self-rated health was assessed on a five-point scale with the following question: In general, how would you rate your health today? The response categories were: ‘very good’, ‘good’, ‘moderate’, ‘bad’ and ‘very bad’. In the analysis, ‘bad’ and ‘very bad’ health categories were combined to represent poor self-rated health.

#### Cognitive score index

To understand the composite effect of cognition we made a cognitive index combining four variables: verbal fluency, verbal recall, digit span forward and digit span backward.

Different cognition tests and procedure used in the survey are;

**Verbal recall**: Interviewer read out a list of 10 commonly used words to the respondents and asked them to repeat again in some time.
**Digit span (forward and backward)**: Participants were read a series of digits and asked to immediately repeat them back. In the backward test, the person must repeat the numbers in reverse order. These tests measure concentration, attention, and immediate memory.
**Verbal fluency**: Participants were asked to produce as many animal names as possible in one-minute time span. This test assessed retrieval of information from semantic memory.

The composite index was derived using Principal Components Analysis (PCA), a mathematical tool which helps in creating a composite index using uncorrelated components, where each component captures the largest possible variation in the original variables. Selected raw scores for cognitive tasks were bundled into three domains (digit span, memory and executive functioning) to yield compound cognitive scores. This was done to condense the number of cognitive variables while refining the robustness of the underlying cognitive construct [52]. We followed two steps to make a cognitive index:

Step 1: All four variables were in different scales. So first, we standardized these variables. A standardized variable (sometimes called a z-score or a standard score) is a variable that has been rescaled to have a mean of zero and a standard deviation of one. Each case's value on the standardized variable designates its difference from the mean of the primary variable in some standard deviations (of the original variable).
Step 2: PCA is a multivariate statistical technique used for extracting from a set of variables those few orthogonal linear combinations that capture the common information most successfully [53]. Further, this index comprises both values, positive and negative. So we converted this index into a 0–100 scale which facilitates easier interpretation of the data. Higher scores indicate better cognitive abilities.

#### Quality of life index (WHO-QoL)

We used the quality of life questionnaire (S-QoL 30) from the WHO-SAGE data set, which was a particular, self-administered and multidimensional QoL questionnaire designed for people. It included 30 items describing five dimensions: physical health, psychological health, level of independence, social relation and environment. It also included a total score (Index). The five dimensions and the Index score ranged from 0 to 100; higher scores indicated better quality of life [49, 54].

### Covariates

The selected demographic and health risk factors were: age (50–59, 60–64, 65–69, 70–79, and ≥80), sex (male and female), place of residence (urban and rural), marital status (currently married and otherwise). We have also included self-reported sleep problems, low back pain, chronic diseases such as hypertension, diabetes, angina, stroke, arthritis and asthma as risk factors of general health and quality of life [18, 28, 55, 56, 57].

Sleep problems: Presence of insomnia symptoms such as difficulty in falling asleep, difficulty staying asleep, or early wakening were assessed in WHO-SAGE survey with the following question. ‘Overall in the last 30 days, how much of a problem did you have with sleeping, such as falling asleep, waking up frequently during the night or waking up too early in the morning?’ The responses were: ‘none’, ‘mild’, ‘moderate’, ‘severe’ and ‘extreme/cannot do’. We combined ‘severe’ and ‘extreme/cannot do’ to represent sleep problems.

In SAGE Survey, the self-reported prevalence of chronic diseases and back pain were assessed through following questions;

1. Have you ever been diagnosed with high blood pressure (hypertension)? (Yes, No)
2. Have you ever been diagnosed with diabetes (high blood sugar)? (Yes, No)
3. Have you ever been diagnosed with angina or angina pectoris (a heart disease)? (Yes, No)
4. Have you ever been told by a health professional that you have had a stroke? (Yes, No)
5. Have you ever been diagnosed with/told you have arthritis (a disease of the joints, or by other names rheumatism or osteoarthritis)? (Yes, No)
6. Have you ever been diagnosed with asthma (an allergic respiratory disease)? (Yes, No)
7. Have you experienced back pain during the last 30 days? (Yes, No)

We also included state/province variable as India is experiencing regional variations in socioeconomic development, demographic and health transition [58, 59, 60]. In SAGE survey, six states were included which consists of Assam, Karnataka, Maharashtra, Rajasthan, Uttar Pradesh and West Bengal.

### Statistical analysis

First, descriptive statistics were calculated by different BMI categories, demographic and socioeconomic variables (Table 1). Second, bivariate analysis was carried out to understand the prevalence of poor self-rated health, mean cognition score and quality of life score by body mass index category.

**Table 1:** Sociodemographic characteristics of the study population by body mass index category (Weighted %).

Characteristics	BMI (Kg/M^2^) Category			
	<18.5 (Underweight)	18.5–24.9 (Normal Weight)	25.0–29.9 (Overweight)	>30.0 (Obesity)
Age group				
50–59	33.7	50.3	13.0	2.8
60–64	39.6	48.1	9.8	2.3
65–69	39.3	50.0	7.8	2.7
70–79	49.0	41.0	7.7	2.1
80+	55.8	36.0	4.4	3.6
Sex				
Male	39.9	50.1	8.0	1.8
Female	37.6	45.5	13.2	3.6
Residence				
Urban	28.6	49.1	18.4	3.7
Rural	42.8	47.3	7.4	2.3
Marital status				
Currently married	37.0	49.7	10.7	2.6
Otherwise	45.2	41.8	10.0	3.0
Wealth quintile				
Lowest	56.1	40.2	2.5	1.1
Second	46.6	44.0	8.1	1.1
Middle	42.0	46.6	9.3	1.8
Fourth	32.9	54.7	10.3	1.9
Highest	21.9	51.8	19.8	6.3
Schooling (Years)				
No schooling	46.3	44.5	6.8	2.3
1–5 years	36.8	47.2	13.6	2.2
6–9 years	29.4	56.3	12.5	1.6
10 years or above	22.2	54.9	17.1	5.6
State				
Assam	41.0	50.9	6.2	1.8
Karnataka	28.6	50.8	16.4	4.1
Maharashtra	31.5	55.3	11.2	1.7
Rajasthan	33.7	49.9	12.5	3.7
Uttar Pradesh	46.9	42.1	8.4	2.4
West Bengal	42.2	45.3	9.5	2.9
Total	38.2	48.2	10.7	2.7

#### Multivariate regression analysis

The study was carried out with a multinomial logistic regression for simultaneous examination of differentials and determinants of body mass index by demographic and socioeconomic characteristics. Multinomial logistic regression is an often used standard statistical tool, particularly when more than four discrete outcomes are needed (such as normal weight, underweight, overweight and obesity). In the first stage of this model, we estimated beta coefficients for the four discrete outcomes by taking ‘normal weight’ as the reference category. The mathematical form of multinomial regression models can be written as:

$$Z_{1}=Log(\frac{{(P}_{1}=Underweight)}{{(P}_{2}=Normal\;Weight(ref))})=\beta_{01}+\beta_{11}{\left(Age\;Group\right)}_{i}+\beta_{12}{\left(Sex\right)}_{i}+\beta_{13}{\left(Residence\right)}_{i}+\beta_{14}{\left(Wealth\;Quantile\right)}_{i}+\beta_{15}{\left(Years\;of\;Schooling\right)}_{i}+\beta_{16}{\left(States\right)}_{i}$$

$$Z_{2}=Log\left(\frac{(P_{3}=Overweight}{\left(P_{2}=Normal\;Weight\left(\text{ref}\right)\right)}\right)=\beta_{02}+\beta_{21}{\left(Age\;Group\right)}_{i}+\beta_{22}{\left(Sex\right)}_{i}+\beta_{23}{\left(Residence\right)}_{i}+\beta_{24}{\left(Wealth\;Quantile\right)}_{i}+\beta_{25}{\left(Years\;of\;Schooling\right)}_{i}+\beta_{26}{\left(States\right)}_{i}$$

$$Z_{3}=Log(\frac{{(P}_{4}=Obesity)}{{(P}_{2}=Normal\;Weight(ref))})=\beta_{03}+\beta_{31}{\left(Age\;Group\right)}_{i}+\beta_{32}{\left(Sex\right)}_{i}+\beta_{33}\;{\left(Residence\right)}_{i}+\beta_{34}{\left(Wealth\;Quantile\right)}_{i}+\beta_{35}{\left(Years\;of\;Schooling\right)}_{i}+\beta_{36}{\;(States)}_{i}$$

Where

*β*_01_ *to β*_03_: *Constant*

*β*_11_ *to β*_36_: *Multinomial Regression Coefficient and ref* = *Reference*

Further, binary logistic regression was used to assess the association of underweight with poor self-rated health with covariates on socio-demographic characteristics, health markers, low back pain, sleep problems and state/province. The binary response (0 = good health and 1 = poor health) for each was related to a set of categorical predictors, X (BMI, age group, sex, residence, marital status, years of schooling, wealth quintile, sleep problems, health markers and state/province).

The logit model was formulated for analysis as:

$$logit\left(\pi_{ij})=log[\pi_{i}/\left(1-\pi_{i}\right)\right]=\beta_{0}+\beta (X)+\varepsilon$$

We used different models to better understand the association of each covariate with poor self-rated health (see S2 File). Model 1, includes only BMI; model 2, includes demographic variables; model 3, includes socioeconomic variables; model 4, includes self-reported sleep problems, chronic diseases and low back pain; model 5, includes state/province variable.

Finally, we used the linear regression model [61] to examine the association of underweight with cognition and quality of life, which can be specified as:

$${Ln(QoL|Cognitive)}_{i}=\beta_{0}+\beta_{1}A_{i}+\beta_{2}B_{i}+\beta_{3}C_{i}+\beta_{4}D_{i}+\beta_{5}E_{i}+\beta_{6}F_{i}+\beta_{7}G_{i}+\beta_{8}H_{i}+\beta_{9}J_{i}+\beta_{10}K_{i}+u_{i}$$

Where

*β*_0_: *Constant*, *β*_1_ *to β*_10_ *regression coefficient and i = stand for the individual*; also *A*_*i*_ = *BMI*, *B*_*i*_ = *age group*, *C*_*i*_ = *sex*, *D*_*i*_ = *residence*, *E*_*i*_ = *marital status*, *F*_*i*_ = *education level*, *G*_*i*_ = *wealth quintile*, *H*_*i*_ = *sleep problems and J*_*i*_ = *states and K*_*i*_ = *health markers*

We used five different models to better understand the association of each covariate with cognition and quality of life as described previously. All the statistical analysis of this study was performed using STATA version 12.0 (Stata Corp LP, College Station, TX, USA).

## Results

Table 1 presents the characteristics of the study population by BMI category. This study used data on respondents of 50 years and above with a total sample of 6,372. The overall prevalence of underweight was 38.2 percent. The percentages of overweight and obesity in the study population were 10.7 and 2.7 respectively. The sample distribution among different age groups by BMI category indicated that a higher share of older adults were underweight in the 80+ category. More than half of the respondents in the age group 50–59 were in the normal weight category. Compared to women (37.6 percent), a higher proportion of men were in the underweight category (39.9 percent). On the other hand, the distribution of women respondents was higher in the overweight and obesity categories compared to men respondents. In rural areas 42.8 percent older adults were underweight compared to 28.6 percent in urban areas. A higher proportion of older adults in the overweight and obesity categories was from urban areas. The distribution of underweight varied considerably by marital status. Increase in years of schooling and wealth associated with lower prevalence of underweight.

**Fig 1:**
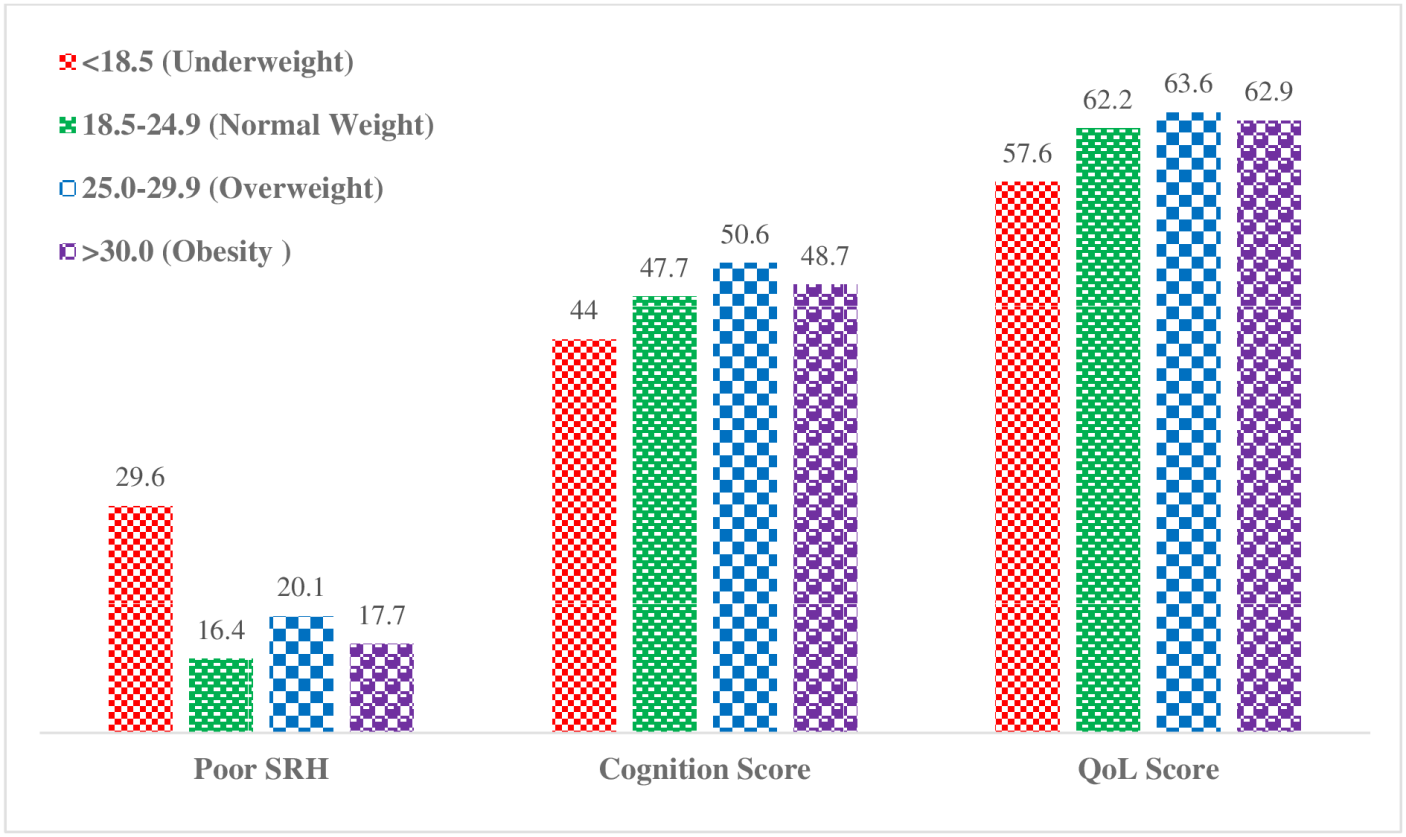
Weighted prevalence of poor self-rated health (SRH), mean cognition and quality of life (QoL) scores by body mass index category.

Fig 1 presents the prevalence of poor self-rated health, mean cognition score and quality of life score by BMI category. The prevalence of poor self-rated health was higher among older adults in the underweight category (29.6 percent) than normal weight (16.4 percent), overweight (20.1 percent) and obesity categories (17.7 percent). Similarly, the mean cognition and quality of life scores were lower among underweight older adults than normal, overweight and obese older adults.

The results of multinomial regression analysis is shown in Table 2 by coefficient and level of significance. According to the table, the probability of being underweight or obese compared to normal weight increases with age. Compared to men, older women were more likely to be obese (β = 3.66; *p* < .001). Further, older adults in rural areas were more likely to be underweight than the urban counterparts (β = 1.44; *p* < .005). It was also evident that the older adults belonging to highest wealth quintile were less likely to be underweight (*p* < .001) compared to those from the lowest wealth quintile (*p* < .001). Further, years of schooling was positively associated with overweight and obesity. Older adults in Uttar Pradesh were more likely to be underweight as compared to the reference group Assam, whereas older adults in Rajasthan were more likely to be obese.

**Table 2:** Multinomial logistic regression coefficient and confidence interval of BMI by socioeconomic and demographic characteristics.

Background	18.5–24.9^(ref)^ (Normal Weight)	<18.5 (Underweight)		25.0–29.9 (Overweight)		>30.0 (Obesity)	
		β	95% CI	β	95% CI	β	95% CI
Age group							
50-59^(ref)^							
60–64		1.14**	(0.97, 1.34)	0.75	(0.59, 0.95)	0.84*	(0.56, 1.26)
65–69		1.26**	(1.06, 1.48)	0.68**	(0.53, 0.88)	0.79*	(0.50, 1.23)
70–79		1.59***	(1.36, 1.88)	0.75	(0.58, 0.96)	0.94**	(0.59, 1.46)
80+		2.08***	(1.58, 2.74)	0.56	(0.33, 0.96)	1.28**	(0.64, 2.53)
Sex							
Male^(ref)^							
Female		0.91	(0.80, 1.03)	2.07**	(1.69, 2.52)	3.66***	(2.48, 5.39)
Residence							
Urban^(ref)^							
Rural		1.44**	(1.24, 1.68)	0.65	(0.54, 0.78)	0.73*	(0.11, 0.99)
Years of schooling							
No schooling^(ref)^							
1–5 years		0.84**	(0.73, 0.97)	1.71	(1.36, 2.14)	1.67**	(1.11, 2.49)
6–9 years		0.62*	(0.48, 0.77)	1.53*	(1.12, 2.07)	0.96*	(0.49, 1.86)
10 years or above		0.52***	(0.40, 0.64)	1.78**	(1.33, 2.37)	2.59**	(1.56, 4.27)
Wealth quintile							
Lowest^(ref)^							
Second		0.73***	(0.61, 0.87)	1.25	(0.80, 1.94)	0.86***	(0.42, 1.75)
Middle		0.62*	(0.51, 0.74)	1.97	(1.30, 2.96)	1.29**	(0.67, 2.45)
Fourth		0.46***	(0.38, 0.55)	2.02**	(1.34, 3.02)	1.08**	(0.57, 2.02)
Highest		0.34***	(0.41, 0.64)	3.16***	(2.11, 4.71)	2.31***	(1.26, 4.23)
State							
Assam^(ref)^							
Karnataka		0.73***	(0.57, 0.92)	2.20***	(1.49, 3.25)	2.38	(1.16, 4.84)
Maharashtra		0.72**	(0.57, 0.90)	1.89	(1.29, 2.78)	1.62	(0.78, 3.33)
Rajasthan		0.75*	(0.60, 0.93)	2.11	(1.43, 3.09)	2.61***	(1.29, 5.27)
Uttar Pradesh		1.22**	(0.98, 1.51)	1.72	(1.15, 2.55)	2.53*	(1.24, 5.15)
West Bengal		0.91**	(0.72, 1.12)	1.76	(1.19, 2.58)	1.81*	(0.88, 3.73)
Sample Size = 6332		Pseudo R^2^ = 0.0844					

β = coefficient value; ref = reference;

*** Significant at p < .001,

** Significant at p < .005,

* Significant at p < .01

Table 3 presents the association of underweight with poor self-rated health, cognition and quality of life after adjusting for relevant covariates. The association of underweight with all outcomes were significant. Compared with normal weight, older adults in the underweight category were 1.60 (*p* < .001) times more likely to report poor self-rated health. Similarly, underweight older adults experienced reduced cognition (β = -0.95; *p* < .001) and quality of life scores (β = -1.90; *p* < .001) compared to normal weight counterparts. Further, the association between education and self-rated health was significant. Demographic factors such as age, female gender and rural residence had negative association with the cognition score. Older adults in currently not married category had lower cognition and quality of life scores. The association of socioeconomic status and cognition were stronger; especially older adults who had 10 or above years of schooling had a score 13.97 (*p* < .001) points higher than older adults with no formal schooling. Similarly, wealth quintile was strongly associated with quality of life; older adults in the highest wealth quintile category had a score 8.41 (*p* < .001) points higher than older adults in the poorest wealth quintile category. Self-reported chronic diseases and low back pain were strongly associated with poor self-rated health and quality of life. The negative association of sleep problems with all three outcomes was significant and stronger.

**Table 3:** Multivariable regression analysis of the association of underweight with poor self-rated health, cognition and quality of life.

Background	Poor self-rated health		Cognition		Quality of Life	
	OR	95% CI	β	95% CI	β	95% CI
BMI						
Underweight	1.60***	(1.37, 1.87)	-0.95***	(-1.46, -0.45)	-1.90***	(-2.52, -1.29)
Normal^(ref)^						
Overweight	1.01	(0.77, 1.31)	1.30***	(0.56, 2.04)	0.33	(-0.56, 1.24)
Obese	1.06	(0.69, 1.63)	0.60	(-0.67, 1.89)	-0.06	(-1.63, 1.49)
Age group						
50-59^(ref)^						
60–64	1.22**	(1.00, 1.50)	-1.09***	(-1.71, -0.47)	-0.95**	(-1.71, -0.20)
65–69	1.51***	(1.23, 1.85)	-1.31***	(-1.96, -0.65)	-1.80***	(-2.60, -1.00)
70–79	2.19***	(1.79, 2.68)	-2.86***	(-3.54, -2.19)	-2.91***	(-3.72, -2.09)
80+	2.81***	(2.07, 3.82)	-4.86***	(-6.02, -3.69)	-4.74***	(-6.11, -3.37)
Sex						
Male^(ref)^						
Female	0.99	(0.84, 1.18)	-3.43***	(-3.96, -2.89)	-0.54	(-1.19, 0.10)
Residence						
Urban^(ref)^						
Rural	1.07	(0.88, 1.29)	-0.48*	(-1.05, 0.07)	0.67*	(-0.01, 1.36)
Marital status						
Currently married^(ref)^						
Otherwise	1.13	(0.95, 1.33)	-1.11***	(-1.67, -0.54)	-1.21***	(-1.89, -0.52)
Years of schooling						
No schooling^(ref)^						
1–5 years	0.78***	(0.65, 0.94)	6.25***	(5.66, 6.84)	1.25***	(0.53, 1.97)
6–9 years	0.61***	(0.45, 0.82)	9.86***	(9.00, 10.7)	2.98***	(1.93, 4.03)
10 years or above	0.54***	(0.40, 0.73)	13.97***	(13.1, 14.8)	5.28***	(4.27, 6.29)
Wealth quintile						
Lowest^(ref)^						
Second	1.04	(0.83, 1.29)	1.12***	(0.36, 1.89)	1.91***	(0.99, 2.83)
Middle	1.04	(0.83, 1.30)	2.02***	(1.25, 2.79)	3.75***	(2.81, 4.68)
Fourth	0.93	(0.73, 1.18)	2.51***	(1.74, 3.29)	5.00***	(4.06, 5.94)
Highest	0.78*	(0.61, 1.01)	3.92***	(3.11, 4.74)	8.41***	(7.42, 9.40)
Health markers (Ref: No)						
Sleep problems	4.37***	(3.68, 5.19)	-1.39***	(-2.07, -0.71)	-7.09***	(-7.91, -6.27)
Hypertension	1.48***	(1.23, 1.78)	-0.24	(-0.87, 0.37)	-1.63***	(-2.39, -0.87)
Diabetes	1.31*	(0.99, 1.73)	0.89**	(0.01, 1.78)	-1.08**	(-2.16, -0.003)
Angina	1.19	(0.88, 1.62)	0.19	(-0.85, 1.24)	-0.73	(-2.02, 0.54)
Stroke	1.28	(0.84, 1.95)	0.39	(-1.18, 1.98)	-2.97***	(-4.83, -1.10)
Arthritis	1.71***	(1.44, 2.04)	-1.28***	(-1.88, -0.67)	-2.40***	(-3.13, -1.66)
Asthma	2.01***	(1.58, 2.54)	-0.07	(-0.96, 0.81)	-4.65***	(-5.72, -3.58)
Back pain	1.76***	(1.52, 2.04)	0.01	(-0.46, 0.49)	-2.85***	(-3.43, -2.27)
State						
Assam^(ref)^						
Karnataka	0.16***	(0.11, 0.21)	0.71	(-0.23, 1.66)	5.25***	(4.09, 6.41)
Maharashtra	0.31***	(0.23, 0.40)	1.75***	(0.85, 2.65)	5.43***	(4.33, 6.54)
Rajasthan	0.29***	(0.22, 0.38)	1.42***	(0.54, 2.30)	5.03***	(3.96, 6.10)
Uttar Pradesh	0.55***	(0.43, 0.71)	0.96**	(0.09, 1.83)	7.08***	(6.02, 8.14)
West Bengal	0.97	(0.76, 1.23)	0.06	(-0.81, 0.95)	-2.89***	(-3.97, -1.81)
Pseudo R^2^	0.1909					
Adjusted R^2^			0.4086		0.3257	
Sample Size	6330		6164		6330	

OR = Odds Ratio; β = coefficient value; CI = Confidence Interval; ref = reference;

*** Significant at p < .001,

** Significant at p < .005,

* Significant at p < .01

## Discussion and conclusion

This study examined the socioeconomic patterns of underweight among older adults aged ≥50 in India. Subsequently, we studied the association of underweight with poor self-rated health, cognition and quality of life outcomes. From this nationally representative and cross-sectional data, we found a strong socioeconomic differential in underweight among older adults aged ≥50. Although, socioeconomic improvement is evident in India in recent decades, a larger proportion of older adults is underweight, especially those of low socioeconomic status. Underweight older adults were more likely to report poor self-rated health and had poor cognition and quality of life scores after adjusting for age, sex, place of residence, marital status, years of schooling, wealth quintile, sleep problems, low back pain, chronic diseases and state.

In this study, a large proportion (38 percent) of older adults aged 50 years and above are underweight. The result is consistent with previous literature which showed higher prevalence of nutritional deficiency among older population in South India [62]. Further, socioeconomic status was found to be strongly associated with underweight, as shown in previous studies conducted among younger and older adults [9, 32, 33, 34, 35, 36, 37]. A higher prevalence of underweight was found among less educated and older adults in the lower wealth quintile category. On the other hand, overweight and obesity were concentrated in wealthier and educated older adults, which suggested a dual burden of malnutrition among older adults characterized by socioeconomic status. Literature shows that higher economic status plays an important role in energy intake along with sedentary behaviours causing a higher body mass index [63, 64]. On the other hand, individuals in poor socioeconomic status are exposed to food insecurity, limited food choice, poor life course socioeconomic status and health risk behaviours, which are strongly associated with lower body mass index [65, 66, 67, 68, 69].

Furthermore, age is strongly associated with underweight which is consistent with prior literature [70]. Existing studies show that increasing age is correlated with various physiological and biological changes such as loss of muscle mass, absorption of iron and vitamin, height and body shape, which in turn lead to lower body mass index [71, 72, 73]. In addition, studies also found age as an important risk factor of change in dietary habits and reduced smell, taste and appetite which further may lead to lower food intake and body mass index [62, 74, 75]. Further, poor oral health condition in old age was found to be significant determinant of involuntary weight loss and being underweight [76, 77].

Most of the studies conducted in India and other Asian countries found a strong and positive association of underweight with excess mortality [11, 25, 26]. This study also supported the hypothesis by showing a strong association of underweight with all three outcomes (poor self-rated health, cognition and quality of life). Prior studies have found U shaped relationship between body mass index and poor self-rated health in United States [18]. In this study, being underweight was consistently associated with poor self-rated health across different models which is consistent with prior literature [28] whereas, overweight and obesity were not associated with poor self-rated health among older adults in India.

The association between lower body weight and quality of life was observed in the present study. Existing literature found similar findings. For example, Zhu et al. (2015) showed significantly lower quality of life scores among underweight adult population in China [29]. However, this study found no significant association between obesity and quality of life contrary to the previous literature [78]. Also, underweight older adults had poor cognitive abilities [27]. Similar to this result, Qizilbash et al. (2015) found that underweight during adulthood and old age is associated with increased risks of dementia and cognitive impairment [79]. On the other hand, older adults in overweight category had better cognitive abilities than normal weight counterparts as shown in other studies [80, 81].

Further, measures of socioeconomic status, such as years of schooling and wealth quintile strongly predicted self-rated health, cognition and quality of life. Previous studies conducted in India and other developing countries also had similar findings [82, 83, 84, 85]. Health markers such as sleep problems, chronic morbidities and low back pain strongly associated with self-rated health and quality of life [55, 56, 57].

While most of the developed and developing countries face the challenges of obesity and related health consequences. This study showing a higher prevalence of underweight in a developing country and its significant implications on poor self-rated health, cognition and quality of life is important to be considered from policy perspective. Also, in recent years, India is experiencing a growing problem of overweight and obesity leading to the dual burden of malnutrition. In addition to obesity-related interventions, special attention on diet and potential nutritional interventions should be given to underweight older adults to improve the overall health and quality of life.

### Strengths and limitations

The strength of this study is that it used nationally representative data and therefore the findings can be generalized at the national level. To our knowledge, this is the first study to examine the association of underweight with various health outcomes among older adults in India. The association of underweight with poor self-rated health, cognition and quality of life were statistically significant. Furthermore, this study used a standardized questionnaire in assessing cognition and quality of life, which provides a comparable estimation of developing and developed countries. While most studies used self-reported height and weight to examine mortality and other health consequences of BMI, this study collected height and weight measurements by trained investigators, which is considered as a standard measure.

The results of this study must be interpreted considering a few limitations. Since the study used cross-sectional data, any causal relationship cannot be established. Further, we used self-reported health assessment such as self-rated health and other measures used in the quality of life scale. Self-reported measures are subject to reporting and cultural bias, especially in the context of developing countries [86]. Furthermore, the size of the effect is relatively small (e.g. see R^2^ values) compared to other predictors of self-rated health, quality of life and cognition. Therefore, it is important to note the role of socioeconomic status and health markers in predicting subjective health and quality of life of the older population in India.

In conclusion, the results of this study suggest a significant socioeconomic disparities in body mass index among older population in India. A significant association of underweight with all three outcomes of poor self-rated health, cognition and quality of life were observed, which needs to be considered to improve the quality of aging in India.

## Supporting information

S1 File List of items used for calculation of household wealth, WHO-SAGE India, 2007–8. (DOCX)

S2 File Regression models of underweight with poor self-rated health, cognition and quality of life. (DOCX)
