# Supplementary material for: Socioeconomic patterns of underweight and its association with self-rated health, cognition and quality of life among older adults in India
Source: PLoS One. 2018 Mar 7;13(3):e0193979. doi: 10.1371/journal.pone.0193979 (PMC5841798; doi:10.1371/journal.pone.0193979)
Supplement: S1 File — (DOCX) [file pone.0193979.s001.docx]

S1 List of items used for calculation of household wealth, WHO-SAGE India, 2007-8

| **Question No.** | **Item details** |
| --- | --- |
| q0700 | Can you please tell me how many rooms there are in your home? |
| q0701 | How many chairs are there in your home? |
| q0702 | How many tables are there in your home? |
| q0703 | How many cars are there in your household? |
| q0704 | Does your home have electricity? |
| q0705 | Does anyone in your household have a bicycle? |
| q0706 | Does anyone in your household have a clock? |
| q0707 | Does anyone in your household have a bucket? |
| q0708 | Does anyone in your household have a washing machine for clothes? |
| q0709 | Does anyone in your household have a refrigerator? |
| q0710 | Does anyone in your household have a refrigerator? |
| q0711 | Does anyone in your household have a fixed line telephone? |
| q0712 | Does anyone in your household have a mobile/cellular telephone? |
| q0713 | Does anyone in your household have a television? |
| q0714 | Does anyone in your household have a computer? |
| q0715 | Does anyone in your household have moped/scooter/motorcycle? |
| q0716 | Does anyone in your household have live-stock (cattle only)? |
| q0717 | Does anyone in your household have sewing machine? |
| q0718 | Does anyone in your household have radio/transistor/tape recorder? |
| q0719 | Does anyone in your household have bullock cart? |
