# Supplementary material for: Socioeconomic patterns of underweight and its association with self-rated health, cognition and quality of life among older adults in India
Source: PLoS One. 2018 Mar 7;13(3):e0193979. doi: 10.1371/journal.pone.0193979 (PMC5841798; doi:10.1371/journal.pone.0193979)
Supplement: S2 File — (DOCX) [file pone.0193979.s002.docx]

S2 Regression models of underweight with poor self-rated health, cognition and quality of life

Table 1. Logistic regression models of underweight and poor self-rated health.

| Background | Model 1: BMI Only | | Model 2: Demographic | | Model 3: SES | | Model 4: Health Markers | | Model 5:State/Province | |
| --- | --- | --- | --- | --- | --- | --- | --- | --- | --- | --- |
|  | OR | 95% CI | OR | 95% CI | OR | 95% CI | OR | 95% CI | OR | 95% CI |
| BMI |  |  |  |  |  |  |  |  |  |  |
| Underweight | 1.97*** | (1.72, 2.24) | 1.75*** | (1.53, 2.00) | 1.59*** | (1.39, 1.83) | 1.69*** | (1.45, 1.96) | 1.60*** | (1.37, 1.87) |
| Normal^(ref)^ |  |  |  |  |  |  |  |  |  |  |
| Overweight | 0.82 | (0.65, 1.03) | 0.89 | (0.70, 1.13) | 0.98 | (0.77, 1.24) | 0.90 | (0.70, 1.16) | 1.01 | (0.77, 1.31) |
| Obese | 1.04 | (0.72, 1.52) | 1.01 | (0.69, 1.49) | 1.11 | (0.75, 1.65) | 0.98 | (0.64, 1.49) | 1.06 | (0.69, 1.63) |
| Age group |  |  |  |  |  |  |  |  |  |  |
| 50-59^(ref)^ |  |  |  |  |  |  |  |  |  |  |
| 60-64 |  |  | 1.28*** | (1.07, 1.54) | 1.25** | (1.04, 1.51) | 1.13 | (0.93, 1.38) | 1.22** | (1.00, 1.50) |
| 65-69 |  |  | 1.64*** | (1.36, 1.97) | 1.62*** | (1.34, 1.96) | 1.35*** | (1.11, 1.65) | 1.51*** | (1.23, 1.85) |
| 70-79 |  |  | 2.33*** | (1.95, 2.79) | 2.35*** | (1.96, 2.81) | 1.93*** | (1.59, 2.35) | 2.19*** | (1.79, 2.68) |
| 80+ |  |  | 3.13*** | (2.39, 4.10) | 3.16*** | (2.40, 4.16) | 2.41*** | (1.79, 3.24) | 2.81*** | (2.07, 3.82) |
| Sex |  |  |  |  |  |  |  |  |  |  |
| Male^(ref)^ |  |  |  |  |  |  |  |  |  |  |
| Female |  |  | 1.32*** | (1.15, 1.52) | 1.25*** | (1.08, 1.46) | 1.05 | (0.89, 1.24) | 0.99 | (0.84, 1.18) |
| Residence |  |  |  |  |  |  |  |  |  |  |
| Urban^(ref)^ |  |  |  |  |  |  |  |  |  |  |
| Rural |  |  | 1.40*** | (1.20, 1.65) | 1.15* | (0.97, 1.37) | 1.19* | (0.99, 1.42) | 1.07 | (0.88, 1.29) |
| Marital status |  |  |  |  |  |  |  |  |  |  |
| Currently Married^(ref)^ |  |  |  |  |  |  |  |  |  |  |
| Otherwise |  |  | 1.24*** | (1.07, 1.45) | 1.18** | (1.01, 1.37) | 1.19** | (1.01, 1.40) | 1.13 | (0.95, 1.33) |
| Years of schooling |  |  |  |  |  |  |  |  |  |  |
| No schooling^(ref)^ |  |  |  |  |  |  |  |  |  |  |
| 1-5 years |  |  |  |  | 1.06 | (0.90, 1.24) | 1.01 | (0.85, 1.20) | 0.78*** | (0.65, 0.94) |
| 6-9 years |  |  |  |  | 0.85 | (0.65, 1.11) | 0.86 | (0.65, 1.14) | 0.61*** | (0.45, 0.82) |
| 10 years or above |  |  |  |  | 0.69** | (0.53, 0.91) | 0.75* | (0.57, 1.01) | 0.54*** | (0.40, 0.73) |
| Wealth quintile |  |  |  |  |  |  |  |  |  |  |
| Lowest^(ref)^ |  |  |  |  |  |  |  |  |  |  |
| Second |  |  |  |  | 0.87 | (0.71, 1.05) | 0.93 | (0.75, 1.15) | 1.04 | (0.83, 1.29) |
| Middle |  |  |  |  | 0.86 | (0.70, 1.05) | 0.89 | (0.71, 1.10) | 1.04 | (0.83, 1.30) |
| Fourth |  |  |  |  | 0.65*** | (0.52, 0.80) | 0.69*** | (0.55, 0.86) | 0.93 | (0.73, 1.18) |
| Highest |  |  |  |  | 0.55*** | (0.44, 0.69) | 0.61*** | (0.47, 0.77) | 0.78* | (0.61, 1.01) |
| Health markers (Ref: No) |  |  |  |  |  |  |  |  |  |  |
| Sleep problems |  |  |  |  |  |  | 4.53*** | (3.84, 5.34) | 4.37*** | (3.68, 5.19) |
| Hypertension |  |  |  |  |  |  | 1.64*** | (1.37, 1.97) | 1.48*** | (1.23, 1.78) |
| Diabetes |  |  |  |  |  |  | 1.15 | (0.88, 1.50) | 1.31* | (0.99, 1.73) |
| Angina |  |  |  |  |  |  | 1.03 | (0.76, 1.39) | 1.19 | (0.88, 1.62) |
| Stroke |  |  |  |  |  |  | 1.55** | (1.03, 2.34) | 1.28 | (0.84, 1.95) |
| Arthritis |  |  |  |  |  |  | 1.62*** | (1.37, 1.91) | 1.71*** | (1.44, 2.04) |
| Asthma |  |  |  |  |  |  | 1.85*** | (1.47, 2.32) | 2.01*** | (1.58, 2.54) |
| Back pain |  |  |  |  |  |  | 1.40*** | (1.22, 1.61) | 1.76*** | (1.52, 2.04) |
| State |  |  |  |  |  |  |  |  |  |  |
| Assam^(ref)^ |  |  |  |  |  |  |  |  |  |  |
| Karnataka |  |  |  |  |  |  |  |  | 0.16*** | (0.11, 0.21) |
| Maharashtra |  |  |  |  |  |  |  |  | 0.31*** | (0.23, 0.40) |
| Rajasthan |  |  |  |  |  |  |  |  | 0.29*** | (0.22, 0.38) |
| Uttar Pradesh |  |  |  |  |  |  |  |  | 0.55*** | (0.43, 0.71) |
| West Bengal |  |  |  |  |  |  |  |  | 0.97 | (0.76, 1.23) |
| Pseudo R^2^ | 0.0198 |  | 0.0514 |  | 0.0603 |  | 0.1471 |  | 0.1909 |  |
| Sample Size | 6372 |  | 6372 |  | 6334 |  | 6330 |  | 6330 |  |

OR= Odds Ratio; CI=Confidence Interval, SES= socioeconomic status, ref=reference, *** Significant at p < .001, ** Significant at p < .005, * Significant at p < .01,

Table 2. Linear regression models of underweight and cognition.

| Background | Model 1: BMI Only | | Model 2: Demographic | | Model 3: SES | | Model 4: Health Markers | | Model 5:State/Province | |
| --- | --- | --- | --- | --- | --- | --- | --- | --- | --- | --- |
|  | β | 95% CI | β | 95% CI | β | 95% CI | β | 95% CI | β | 95% CI |
| BMI |  |  |  |  |  |  |  |  |  |  |
| Underweight | -3.90*** | (-4.52, -3.29) | -2.73*** | (-3.29, -2.17) | -1.00*** | (-1.51, -0.50) | -1.02*** | (-1.52, -0.51) | -0.95*** | (-1.46, -0.45) |
| Normal^(ref)^ |  |  |  |  |  |  |  |  |  |  |
| Overweight | 3.15*** | (2.24, 4.06) | 3.07*** | (2.24, 3.90) | 1.32*** | (0.58, 2.05) | 1.37*** | (0.63, 2.11) | 1.30*** | (0.56, 2.04) |
| Obese | 1.62** | (0.01, 3.22) | 2.81*** | (1.36, 4.27) | 0.65 | (-0.63, 1.94) | 0.65 | (-0.63, 1.94) | 0.60 | (-0.67, 1.89) |
| Age group |  |  |  |  |  |  |  |  |  |  |
| 50-59^(ref)^ |  |  |  |  |  |  |  |  |  |  |
| 60-64 |  |  | -1.89*** | (-2.60, -1.19) | -1.13*** | (-1.75, -0.51) | -1.05*** | (-1.67, -0.43) | -1.09*** | (-1.71, -0.47) |
| 65-69 |  |  | -2.44*** | (-3.18, -1.70) | -1.43*** | (-2.09, -0.78) | -1.27*** | (-1.93, -0.61) | -1.31*** | (-1.96, -0.65) |
| 70-79 |  |  | -4.12*** | (-4.87, -3.36) | -3.00*** | (-3.67, -2.33) | -2.82*** | (-3.50, -2.14) | -2.86*** | (-3.54, -2.19) |
| 80+ |  |  | -6.64*** | (-7.95, -5.32) | -5.11*** | (-6.27, -3.94) | -4.85*** | (-6.02, -3.68) | -4.86*** | (-6.02, -3.69) |
| Sex |  |  |  |  |  |  |  |  |  |  |
| Male^(ref)^ |  |  |  |  |  |  |  |  |  |  |
| Female |  |  | -7.72*** | (-8.26, -7.18) | -3.69*** | (-4.21, -3.16) | -3.49*** | (-4.03, -2.96) | -3.43*** | (-3.96, -2.89) |
| Residence |  |  |  |  |  |  |  |  |  |  |
| Urban^(ref)^ |  |  |  |  |  |  |  |  |  |  |
| Rural |  |  | -4.49*** | (-5.08, -3.90) | -0.65** | (-1.20, -0.10) | -0.59** | (-1.14, -0.04) | -0.48* | (-1.05, 0.07) |
| Marital status |  |  |  |  |  |  |  |  |  |  |
| Currently Married^(ref)^ |  |  |  |  |  |  |  |  |  |  |
| Otherwise |  |  | -2.07*** | (-2.71, -1.42) | -1.18*** | (-1.75, -0.62) | -1.19*** | (-1.75, -0.62) | -1.11*** | (-1.67, -0.54) |
| Years of schooling |  |  |  |  |  |  |  |  |  |  |
| No schooling^(ref)^ |  |  |  |  |  |  |  |  |  |  |
| 1-5 years |  |  |  |  | 5.98*** | (5.41, 6.55) | 6.02*** | (5.44, 6.59) | 6.25*** | (5.66, 6.84) |
| 6-9 years |  |  |  |  | 9.67*** | (8.82, 10.5) | 9.64*** | (8.80, 10.49) | 9.86*** | (9.00, 10.7) |
| 10 years or above |  |  |  |  | 13.7*** | (12.9, 14.5) | 13.65*** | (12.8, 14.5) | 13.97*** | (13.1, 14.8) |
| Wealth quintile |  |  |  |  |  |  |  |  |  |  |
| Lowest^(ref)^ |  |  |  |  |  |  |  |  |  |  |
| Second |  |  |  |  | 1.26*** | (0.50, 2.02) | 1.19*** | (0.43, 1.95) | 1.12*** | (0.36, 1.89) |
| Middle |  |  |  |  | 2.15*** | (1.39, 2.92) | 2.11*** | (1.35, 2.88) | 2.02*** | (1.25, 2.79) |
| Fourth |  |  |  |  | 2.82*** | (2.06, 3.59) | 2.72*** | (1.95, 3.49) | 2.51*** | (1.74, 3.29) |
| Highest |  |  |  |  | 4.31*** | (3.51, 5.11) | 4.14*** | (3.34, 4.94) | 3.92*** | (3.11, 4.74) |
| Health markers (Ref: No) |  |  |  |  |  |  |  |  |  |  |
| Sleep problems |  |  |  |  |  |  | -1.51*** | (-2.19, -0.82) | -1.39*** | (-2.07, -0.71) |
| Hypertension |  |  |  |  |  |  | -0.39 | (-1.02, 0.23) | -0.24 | (-0.87, 0.37) |
| Diabetes |  |  |  |  |  |  | 0.98** | (0.09, 1.86) | 0.89** | (0.01, 1.78) |
| Angina |  |  |  |  |  |  | 0.20 | (-0.84, 1.25) | 0.19 | (-0.85, 1.24) |
| Stroke |  |  |  |  |  |  | 0.23 | (-1.34, 1.81) | 0.39 | (-1.18, 1.98) |
| Arthritis |  |  |  |  |  |  | -1.40*** | (-2.00, -0.81) | -1.28*** | (-1.88, -0.67) |
| Asthma |  |  |  |  |  |  | -0.004 | (-0.89, 0.88) | -0.07 | (-0.96, 0.81) |
| Back pain |  |  |  |  |  |  | 0.12 | (-0.34, 0.59) | 0.01 | (-0.46, 0.49) |
| State |  |  |  |  |  |  |  |  |  |  |
| Assam^(ref)^ |  |  |  |  |  |  |  |  |  |  |
| Karnataka |  |  |  |  |  |  |  |  | 0.71 | (-0.23, 1.66) |
| Maharashtra |  |  |  |  |  |  |  |  | 1.75*** | (0.85, 2.65) |
| Rajasthan |  |  |  |  |  |  |  |  | 1.42*** | (0.54, 2.30) |
| Uttar Pradesh |  |  |  |  |  |  |  |  | 0.96** | (0.09, 1.83) |
| West Bengal |  |  |  |  |  |  |  |  | 0.06 | (-0.81, 0.95) |
| Adjusted R^2^ | 0.0426 |  | 0.2241 |  | 0.4024 |  | 0.4063 |  | 0.4086 |  |
| Sample Size | 6206 |  | 6206 |  | 6168 |  | 6164 |  | 6164 |  |

β= coefficient value, CI= Confidence Interval, SES=socioeconomic status, ref= reference, *** Significant at p < .001, ** Significant at p < .005, * Significant at p < .01

Table 3. Linear regression models of underweight and quality of life.

| Background | Model 1: BMI Only | | Model 2: Demographic | | Model 3: SES | | Model 4: Health Markers | | Model 5:State/Province | |
| --- | --- | --- | --- | --- | --- | --- | --- | --- | --- | --- |
|  | β | 95% CI | β | 95% CI | β | 95% CI | β | 95% CI | β | 95% CI |
| BMI |  |  |  |  |  |  |  |  |  |  |
| Underweight | -4.56*** | (-5.26, -3.86) | -3.68*** | (-4.38, -2.99) | -1.93*** | (-2.61, -1.26) | -1.96*** | (-2.60, -1.31) | -1.90*** | (-2.52, -1.29) |
| Normal^(ref)^ |  |  |  |  |  |  |  |  |  |  |
| Overweight | 2.22*** | (1.17, 3.27) | 1.85*** | (0.81, 2.89) | 0.25 | (-0.74, 1.25) | 0.66 | (-0.27, 1.61) | 0.33 | (-0.56, 1.24) |
| Obese | 1.15 | (-0.69, 2.99) | 1.52* | (-0.27, 3.33) | -0.31 | (-2.04, 1.40) | 0.33 | (-1.30, 1.97) | -0.06 | (-1.63, 1.49) |
| Age group |  |  |  |  |  |  |  |  |  |  |
| 50-59^(ref)^ |  |  |  |  |  |  |  |  |  |  |
| 60-64 |  |  | -1.42*** | (-2.29, -0.55) | -1.11*** | (-1.94, -0.27) | -0.56 | (-1.35, 0.22) | -0.95** | (-1.71, -0.20) |
| 65-69 |  |  | -2.91*** | (-3.83, -1.99) | -2.72*** | (-3.60, -1.84) | -1.70*** | (-2.54, -0.87) | -1.80*** | (-2.60, -1.00) |
| 70-79 |  |  | -4.15*** | (-5.09, -3.21) | -4.00*** | (-4.90, -3.11) | -2.60*** | (-3.45, -1.74) | -2.91*** | (-3.72, -2.09) |
| 80+ |  |  | -6.16*** | (-7.73, -4.59) | -6.28*** | (-7.78, -4.77) | -4.45*** | (-5.89, -3.02) | -4.74*** | (-6.11, -3.37) |
| Sex |  |  |  |  |  |  |  |  |  |  |
| Male^(ref)^ |  |  |  |  |  |  |  |  |  |  |
| Female |  |  | -2.73*** | (-3.39, -2.06) | -1.85*** | (-2.55, -1.15) | -0.95*** | (-1.62, -0.27) | -0.54 | (-1.19, 0.10) |
| Residence |  |  |  |  |  |  |  |  |  |  |
| Urban^(ref)^ |  |  |  |  |  |  |  |  |  |  |
| Rural |  |  | -2.17*** | (-2.90, -1.43) | 0.82** | (0.07, 1.56) | 0.80** | (0.09, 1.50) | 0.67* | (-0.01, 1.36) |
| Marital status |  |  |  |  |  |  |  |  |  |  |
| Currently married^(ref)^ |  |  |  |  |  |  |  |  |  |  |
| Otherwise |  |  | -2.58*** | (-3.37, -1.79) | -1.75*** | (-2.50, -0.99) | -1.61*** | (-2.33, -0.90) | -1.21*** | (-1.89, -0.52) |
| Years of schooling |  |  |  |  |  |  |  |  |  |  |
| No schooling^(ref)^ |  |  |  |  |  |  |  |  |  |  |
| 1-5 years |  |  |  |  | -0.44 | (-1.21, 0.32) | -0.27 | (-1.00, 0.45) | 1.25*** | (0.53, 1.97) |
| 6-9 years |  |  |  |  | 1.60*** | (0.47, 2.74) | 1.53*** | (0.45, 2.61) | 2.98*** | (1.93, 4.03) |
| 10 years or above |  |  |  |  | 4.32*** | (3.23, 5.42) | 4.00*** | (2.96, 5.04) | 5.28*** | (4.27, 6.29) |
| Wealth quintile |  |  |  |  |  |  |  |  |  |  |
| Lowest^(ref)^ |  |  |  |  |  |  |  |  |  |  |
| Second |  |  |  |  | 2.73*** | (1.72, 3.75) | 2.42*** | (1.46, 3.37) | 1.91*** | (0.99, 2.83) |
| Middle |  |  |  |  | 4.75*** | (3.73, 5.78) | 4.60*** | (3.63, 5.57) | 3.75*** | (2.81, 4.68) |
| Fourth |  |  |  |  | 6.82*** | (5.80, 7.84) | 6.43*** | (5.46, 7.40) | 5.00*** | (4.06, 5.94) |
| Highest |  |  |  |  | 10.78*** | (9.71, 11.84) | 10.11*** | (9.10, 11.12) | 8.41*** | (7.42, 9.40) |
| Health markers (Ref: No) |  |  |  |  |  |  |  |  |  |  |
| Sleep problems |  |  |  |  |  |  | -7.87*** | (-8.73, -7.01) | -7.09*** | (-7.91, -6.27) |
| Hypertension |  |  |  |  |  |  | -2.37*** | (-3.17, -1.58) | -1.63*** | (-2.39, -0.87) |
| Diabetes |  |  |  |  |  |  | -0.64 | (-1.77, 0.48) | -1.08** | (-2.16, -0.003) |
| Angina |  |  |  |  |  |  | -0.18 | (-1.52, 1.15) | -0.73 | (-2.02, 0.54) |
| Stroke |  |  |  |  |  |  | -4.61*** | (-6.56, -2.67) | -2.97*** | (-4.83, -1.10) |
| Arthritis |  |  |  |  |  |  | -3.09*** | (-3.84, -2.34) | -2.40*** | (-3.13, -1.66) |
| Asthma |  |  |  |  |  |  | -4.17*** | (-5.29, -3.05) | -4.65*** | (-5.72, -3.58) |
| Back pain |  |  |  |  |  |  | -1.98*** | (-2.57, -1.38) | -2.85*** | (-3.43, -2.27) |
| State |  |  |  |  |  |  |  |  |  |  |
| Assam^(ref)^ |  |  |  |  |  |  |  |  |  |  |
| Karnataka |  |  |  |  |  |  |  |  | 5.25*** | (4.09, 6.41) |
| Maharashtra |  |  |  |  |  |  |  |  | 5.43*** | (4.33, 6.54) |
| Rajasthan |  |  |  |  |  |  |  |  | 5.03*** | (3.96, 6.10) |
| Uttar Pradesh |  |  |  |  |  |  |  |  | 7.08*** | (6.02, 8.14) |
| West Bengal |  |  |  |  |  |  |  |  | -2.89*** | (-3.97, -1.81) |
| Adjusted R^2^ | 0.0349 |  | 0.0499 |  | 0.1754 |  | 0.2598 |  | 0.3257 |  |
| Sample Size | 6372 |  | 6372 |  | 6334 |  | 6330 |  | 6330 |  |

β= coefficient value, CI= Confidence Interval, SES= socioeconomic status, ref= reference, *** Significant at p < .001, ** Significant at p < .005, * Significant at p < .01
